# Data for occupancy internal heat gain calculation in main building categories

**DOI:** 10.1016/j.dib.2017.10.036

**Published:** 2017-11-07

**Authors:** Kaiser Ahmed, Jarek Kurnitski, Bjarne Olesen

**Affiliations:** a Aalto University, Department of Civil Engineering, Finland; b Tallinn University of Technology, Faculty of Civil Engineering, Estonia; c Technical University of Denmark, Department of Civil Engineering, Denmark

**Keywords:** Body surface area, Metabolic rate, Dry heat loss, Total heat loss, Internal heat gain

## Abstract

Heat losses from occupant body by means of convection, radiation, vapor, and sweat are essential data for indoor climate and energy simulations. Heat losses depend on the metabolic activity and body surface area. Higher variations of body surface area of occupants are observed in day care centers, kinder gardens and schools compared to other building categories (Tables 2 and 3) and these variations need to be accounted, otherwise in these building categories heat gains, CO_2_ and humidity generation are overestimated. Indoor temperature, humidity level, air velocity, and clothing insulation have significant influences on dry and total heat losses from occupant body leading to typical values for summer and winter. The data presented in this article are related to the research article entitled Occupancy schedules for energy simulation in new prEN16798-1 and ISO/FDIS 17772-1 standards (Ahmed et al., 2017) [1].

**Specification Table**

**Table:** 

Subject area	Construction & Building technology
More specific subject area	Energy and indoor climate
Type of data	Calculated
How data was acquired	Calculated with Equations provided in the research article for main building categories
Data format	Table
Experimental factors	
Experimental features	
Data source location	Research article [1]
Related research article	Occupancy schedules for energy simulation in New prEN16798-1 and ISO/FDIS 17772-1 standards [1].

**Value of the data**

• The data provided in this paper may be used as occupancy related input data for indoor climate and energy simulations in main building categories.
• The data presented the effects of variation of body surface area of occupants in day care center, kinder garden, and school. These differences are to be accounted; otherwise the heat gains are overestimated.
• The data might support the researchers to get information about the heat losses from occupant by means of convection, radiation, vapor and sweat as well as humidity and CO_2_ generation.
• The data provided the information about typical dry and total heat loss values from occupancy in main building categories during summer and winter.

## 1 Data

The data presented in this article are related to the research article entitled Occupancy schedules for energy simulation in new prEN16798-1 and ISO/FDIS 17772-1 standards [1]. The data allow to calculate the dry and total heat losses from occupant body in different building categories during summer and winter. Six parameters affect for estimating the heat losses from occupant body, namely metabolic rate, air temperature, radiant temperature, air velocity, humidity, and clothing insulation.

Activity level (Table 1) and body surface area (Table 2) is the starting point of heat losses calculation of occupant body. Activity level is presented in the form of metabolic rate and depends on the muscular activities. Met is the unit of metabolism that equivalent to 58.15 W/m^2^ per body surface area. According to different building categories, the metabolism rate is illustrated in Table 1.

**Table 1:** Metabolic rates of main building categories [2], [3], [4], [5].

Institution			Metabolic rate (met)
Day care center	Children	2–4 yr	1.0
	Professional	Adult people	1.91
Kinder garden	Children	5–6 yr	1.39
School	Grade 1–6	7–12 yr	1.2
	Grade 7–12	13–18 yr	1.2
	Teacher	Adult people	1.46–1.72
Department store		Adult worker	1.6
Office, Meeting room		Adult office worker (Sedentary)	1.2
Detached house, Apartment building		Adult people	1.2
Hotel, Restaurant, Hospital		Adult people (Sedentary)	1.2
Sport, terminal, theatre		Adult people	1.6

**Table 2:** Body surface area ($A_{\mathrm{DU}}$) of occupants in day care center, kinder garden, school [7].

		Girl/Female					Boy/Male				
	Age	Weight, W		Height, H		Body surface area, $A_{\mathrm{DU}}$	Weight, W		Height, H		Body surface area, $A_{\mathrm{DU}}$
		(lb)	(kg)	(in.)	(m)	(m^2^)	(lb)	(kg)	(in.)	(m)	(m^2^)
Day care center											
	2	26.5	12.0	33.5	0.9	0.52	27.5	12.5	34.2	0.9	0.53
	3	31.5	14.3	37.0	0.9	0.60	31.0	14.1	37.5	1.0	0.60
	4	34.0	15.5	39.5	1.0	0.65	36.0	16.4	40.3	1.0	0.67

Kinder garden											
	5	39.5	18.0	42.5	1.1	0.73	40.5	18.4	43.0	1.1	0.74
	6	44.0	20.0	45.5	1.2	0.80	45.5	20.7	45.5	1.2	0.81

School											
G–1	7	49.5	22.5	47.7	1.2	0.87	50.5	23.0	48.0	1.2	0.88
G–2	8	57.0	25.9	50.5	1.3	0.96	56.5	25.7	50.4	1.3	0.96
G–3	9	62.0	28.2	52.5	1.3	1.03	63.0	28.6	52.5	1.3	1.04
G–4	10	70.5	32.0	54.5	1.4	1.12	70.5	32.0	54.5	1.4	1.12
G–5	11	81.5	37.0	56.7	1.4	1.22	78.5	35.7	56.5	1.4	1.20
G–6	12	91.5	41.6	59.0	1.5	1.32	88.0	40.0	58.7	1.5	1.29
G–7	13	101.0	45.9	61.7	1.6	1.42	100.0	45.5	61.5	1.6	1.41
G–8	14	105.0	47.7	62.5	1.6	1.46	112.0	50.9	64.5	1.6	1.54
G–9	15	115.0	52.3	62.9	1.6	1.52	123.5	56.1	67.0	1.7	1.65
G–10	16	118.0	53.6	64.0	1.6	1.56	134.0	60.9	68.3	1.7	1.73
G–11	17	120.0	54.5	64.0	1.6	1.57	142.0	64.5	69.0	1.8	1.78
G–12	18	125.0	56.8	64.2	1.6	1.60	147.0	66.8	69.2	1.8	1.81
Other		139.9	63.5	65.3	1.6	1.71	159.8	72.5	70.8	1.8	1.91

Body surface area ($A_{\mathrm{DU}}$) is a unique parameter for an individual that depends on the individual height (H) and weight (W). It is considered as the most significant parameter that may responsible for different heat losses from the body, though occupants have the same muscular activity. Also, this is used as an input variable to estimate the heat losses from occupant body by means of convection, radiation, vapor, sweat. The body surface area ($A_{\mathrm{DU}}$) is estimated from the Du Bois formula [6]. Maximum variations of body surface area ($A_{\mathrm{DU}}$) of occupants occur in day care centers, kinder gardens, and schools, shown in Table 2. Other building categories, mostly occupied by the adults, are shown in Table 3.

**Table 3:** The average body surface area ($A_{\mathrm{DU}}$) used in the calculations of occupant heat emission [1].

Building type	Body surface area, $A_{\mathrm{DU}}$ (m^2^)
Detached house	1.80
Apartment building	1.80
Office building	1.80
Department store	1.80
Hotel	1.80
Restaurant	1.80
Sport, terminal, theatre	1.80
School	1.68
Daycare center (2–4 yr)	0.66
Kinder garden (5–6 yr)	0.77
Hospital	1.80

## 2 Materials and method

Seasonal effect are to be accounted to estimate the heat losses from occupant body, because the set point of indoor air temperature as well as humidity levels depend on the seasons. Also, clothing insulation, acceptance of air velocity is changed according to the season. To estimate the heat losses from the body, the input values during summer and winter are presented in the research article, Table 1 [1].

The corresponding heat losses equations are presented in the research article entitled Occupancy schedules for energy simulation in new prEN16798-1 and ISO/FDIS 17772-1 standards [1]. The convection heat losses ($Q_{\mathrm{Convection}}$), radiation heat losses ($Q_{\mathrm{Radiation}}$), vapor heat losses $(Q_{\mathrm{Vapor}})$, and sweat heat losses $(Q_{\mathrm{Sweat}})$ during summer and winter are shown in Table 4, Table 5, respectively.

**Table 4:** Heat loss components of occupancy during summer.

Building type	Metabolic rate (met) [2], [3], [4], [5]	$Q_{\mathrm{Convection}}$ (W)	$Q_{\mathrm{Radiation}}$ (W)	$Q_{\mathrm{Vapor}}$ (W)	$Q_{\mathrm{Sweat}}$ (W)
Detached house	1.2	44.1	38.7	25.9	9.7
Apartment building	1.2	44.1	38.7	25.9	9.7
Office building	1.2	44.1	38.7	25.9	9.7
Department store	1.6	41.7	36.0	27.8	52.4
Hotel	1.2	44.1	38.7	25.9	9.7
Restaurant	1.2	44.1	38.7	25.9	9.7
Sport, terminal, theatre	1.6	41.7	36.0	27.8	52.4
School	1.2	39.6	32.7	25.2	19.4
Daycare center (2–4 yr)	1.0	16.1	13.4	9.5	0.0
Kinder garden (5–6 yr)	1.39	17.6	14.4	11.9	18.1
Hospital	1.2	44.1	38.7	25.9	9.7

**Table 5:** Heat loss components of occupancy during winter.

Building type	Metabolic rate (met) [2], [3], [4], [5]	$Q_{\mathrm{Convection}}$ (W)	$Q_{\mathrm{Radiation}}$ (W)	$Q_{\mathrm{Vapor}}$ (W)	$Q_{\mathrm{Sweat}}$ (W)
Detached house	1.2	38.3	39.5	33.4	7.1
Apartment building	1.2	38.3	39.5	33.4	7.1
Office building	1.2	38.3	39.5	33.4	7.1
Department store	1.6	36.9	37.3	35.9	47.6
Hotel	1.2	38.3	39.5	33.4	7.1
Restaurant	1.2	38.3	39.5	33.4	7.1
Sport, terminal, theatre	1.6	36.9	37.3	35.9	47.6
School	1.2	37.8	38.9	32.9	7.2
Daycare center (2–4 yr)	1.0	15.1	15.8	12.5	0.0
Kinder garden (5–6 yr)	1.39	17.1	17.4	15.7	11.9
Hospital	1.2	38.3	39.5	33.4	7.1

Dry (sensible) heat losses ($Q_{\mathrm{Dry}})$ is the summation of convection heat losses ($Q_{\mathrm{Convection}}$), and radiation heat losses ($Q_{\mathrm{Radiation}}$), whereas total heat losses ($Q_{\mathrm{Total}})$ include all losses from occupant body. The obtained dry and total heat losses are presented in research article [1], which obtained from Table 4, Table 5.

Occupants are the main source of CO_2_ and humidity generation in indoor environment. Metabolic rate and body surface area are the key variables to estimate the CO_2_ and humidity generation [8] and the values for main building categories are shown in Table 6.

**Table 6:** Occupant CO_2_ and humidity generation in main building categories.

Building type	Metabolic rate (met) [2], [3], [4], [5]	Body surface area, $A_{\mathrm{DU}}$ (m^2^) [1]	CO_2_ generation $\left(\frac{l}{h}\right)$	Humidity generation $\left(\frac{g}{h}\right)$	
				Summer	Winter
Detached house	1.2	1.80	18.8	55.7	63.7
Apartment building	1.2	1.80	18.8	55.7	63.7
Office building	1.2	1.80	18.8	55.7	63.7
Department store	1.6	1.80	25.1	125.7	131.2
Hotel	1.2	1.80	18.8	55.7	63.7
Restaurant	1.2	1.80	18.8	55.7	63.7
Sport, terminal, theatre	1.6	1.80	25.1	125.7	131.2
School	1.2	1.68	17.6	52.0	59.3
Daycare center	1.0	0.66	5.8	14.4	18.5
Kinder garden	1.39	0.77	9.3	38.1	40.9
Hospital	1.2	1.80	18.8	55.7	63.7
